# Risk factors for nosocomial rectal colonisation with carbapenem-resistant Gram-negative bacilli in children with haematological malignancies: a case-control study

**DOI:** 10.1186/s12941-023-00622-x

**Published:** 2023-08-03

**Authors:** Chao Fang, Zheng Zhou, Mingming Zhou, Jianping Li

**Affiliations:** Department of Clinical Laboratory, Children’s Hospital, Zhejiang University School of Medicine, National Clinical Research Center for Child Health, No. 3333 Binsheng road, Hangzhou, Zhejiang Province China

**Keywords:** Carbapenem-resistant Enterobacteriaceae, Intestines, Colonisation, Risk factors, Haematologic neoplasms, Child, Case-control studies

## Abstract

**Background:**

Rectal colonisation with carbapenem-resistant Gram-negative bacilli (CR-GNB) may cause CR-GNB infection in children with haematological malignancies (HMs) haematological. To date, information on its epidemiology is limited. This study aimed to assess the the risk factors for rectal colonisation with CR-GNB in children with HMs.

**Methods:**

A case–control study in a tertiary children’s hospital in Hangzhou City, was conducted between July 2019, and September 2021. Based on the hospitalisation date, children in the CR-GNB colonisation group and control groups were matched at a ratio of 1:2. Conditional logistic regression models were used to compute the adjusted odds ratios (aORs) and 95% confidence intervals (CIs) of the risk factors for CR-GNB rectal colonisation in children with HMs.

**Results:**

A total of 85 non-duplicated CR-GNB isolates were collected from rectal swab samples of 69 children with HMs. The 30-day mortality rates were 5.8% in the CR-GNB colonisation group and 0% in the control group (*P* = 0.020).colonisation In the conditional logistic regression model, the aORs were 6.84 (95% CI 1.86–25.20) for acute myeloid leukemia (AML) or acute lymphoblastic leukemia (ALL), 4.16 (95% CI 1.17–14.84) for prior concomitant infections within the last 1 month, 2.33 (95% CI 1.16–4.69) for prior carbapenems usage within the last 1 month and 7.46 (95% CI 1.81–30.67) for prior hematopoietic stem-cell transplantation (HSCT).

**Conclusion:**

AML/ALL, prior concomitant infections within the last 1 month, prior carbapenems usage within the last 1 month, and prior HSCT are associated with an increased risk of rectal colonisation with CR-GNB in children with HMs.

## Background

Carbapenem-resistant Gram-negative bacilli (CR-GNB) mainly include carbapenem-resistant Enterobacteriaceae (CRE), carbapenem-resistant *Pseudomonas* aeruginosa, carbapenem-resistant *Acinetobacter baumannii* and have become serious threats to public health, causing serious infection and high mortality rates [1–4]. CR-GNB-related infectious diseases are becoming increasingly serious and thus have attracted considerable attention. According to the China Antimicrobial Surveillance Network, the prevalence of carbapenem-resistant *K. pneumoniae* has increased from 3% to 2005 to 25.3% in 2019 in Mainland China [5]. It is a marked increase, indicating that CR-GNB-related infectious diseases are becoming increasingly serious and thus have attracted considerable attention. Gut colonisation of CR-GNB is associated with an increased risk of CR-GNB infection and higher mortality rates [4, 6–9]. The US Centers for Disease Control and Prevention and the European Society of Clinical Microbiology and Infectious Diseases also recommended active surveillance of patients at high risk for stool or rectal CRE carriage [10]. Therefore, studies on CR-GNB intestinal colonisation are essential to prevent CR-GNB infections.

Because of the underlying disease, agranulocytosis caused by chemotherapy, and frequent use of antibacterial agents, patients with haematological malignancies (HMs) are predisposed to CR-GNB infections [11–13]. Patients with HMs are also at high risk of CR-GNB infections; thus, the surveillance of patients with HMs for stool or rectal CR-GNB carriage must be strengthened. The risk factors for CRE colonisation or infections in children have been described in previous literature reports [14] However, thus, the surveillance of patients with HMs for stool or rectal CR-GNB carriage must be strengthened. The risk factors for CRE colonisation or infections in children have been described in previous literature reports. Therefore, we aimed to evaluate the risk factors for rectal colonisation in children with HMs and hoped to provide a more reliable strategy for preventing CR-GNB infection in these children.

## Methods

### Setting and study design

This case–control study was performed at Children’s Hospital of Zhejiang University School of Medicine in Hangzhou City. This hospital is the largest tertiary Children’s hospital and the most comprehensive center of pediatric healthcare in Zhejiang Province. The haematology and oncology department of the hospital is one of the earliest pediatric haematology and oncology centers in China and the main unit of Zhejiang Pediatric Leukemia Diagnosis and Treatment Technology Research Center. The study was approved by the ethics committee of the Children’s Hospital, Zhejiang University School of Medicine, China. The need for informed consent was waived because anonymous and de-identified data collected from medical and laboratory records were used.

Since July 2019, the haematology and oncology department (admission was greater than 48 h) has conducted CR-GNB tests on rectal swabs from children with HMs. The direct MacConkey plate method with carbapenem discs was followed for the initial screening of strains in rectal swabs [15]. Identification and further antimicrobial susceptibility testing of suspected strains were performed using Vitek2 Compact (bioMérieux, Marcyl’Étoile, France) following annually published clinical and laboratory standards. Moreover, the Kirby–Bauer disc diffusion method was used to double-check the susceptibility of the suspected strains to the three carbapenems (i.e., imipenem, meropenem and ertapenem). CR-GNB has been defined as an isolate resistant to any of the carbapenem discs except for natural resistance. A retrospective analysis of cases of children with CR-GNB rectal colonisation detected between July 2019 and September 2021 was conducted, and children without HMs were excluded from the analysis. Only relevant information from the first episode was collected if more than one episode of CR-GNB colonisation occurred in the same children. In this study, data collected from the medical records included age, sex, underlying disease, prior concomitant infections within the last 1 month, prior antibiotic use within the last 1 month and prior chemotherapy history. The results of routine blood tests and C-reactive protein (CRP) taken within 48 h of the index date were obtained from the laboratory records. Blood routine and CRP tests were evaluated by BC-5310CRP (Mindray, China). The sample size estimation was based on the preliminary results of the univariate analysis and was performed using the conditional logistic regression module of PASS 15.0.5 (NCSS LLC, Kaysville, UT, USA). The analysis results showed that at least 195 cases should be included in this study after accounting for a 20% dropout rate (power, 0.9; alpha, 0.05). During the study period, a total of 376 children with HMs were tested for rectal CR-GNB, and CR-GNB isolates were isolated from rectal swab samples of 69 children. Because CR-GNB rectal colonisation was mainly interfered with by the hospital environment, the children included in the study were all from the same ward.

**Fig. 1:**
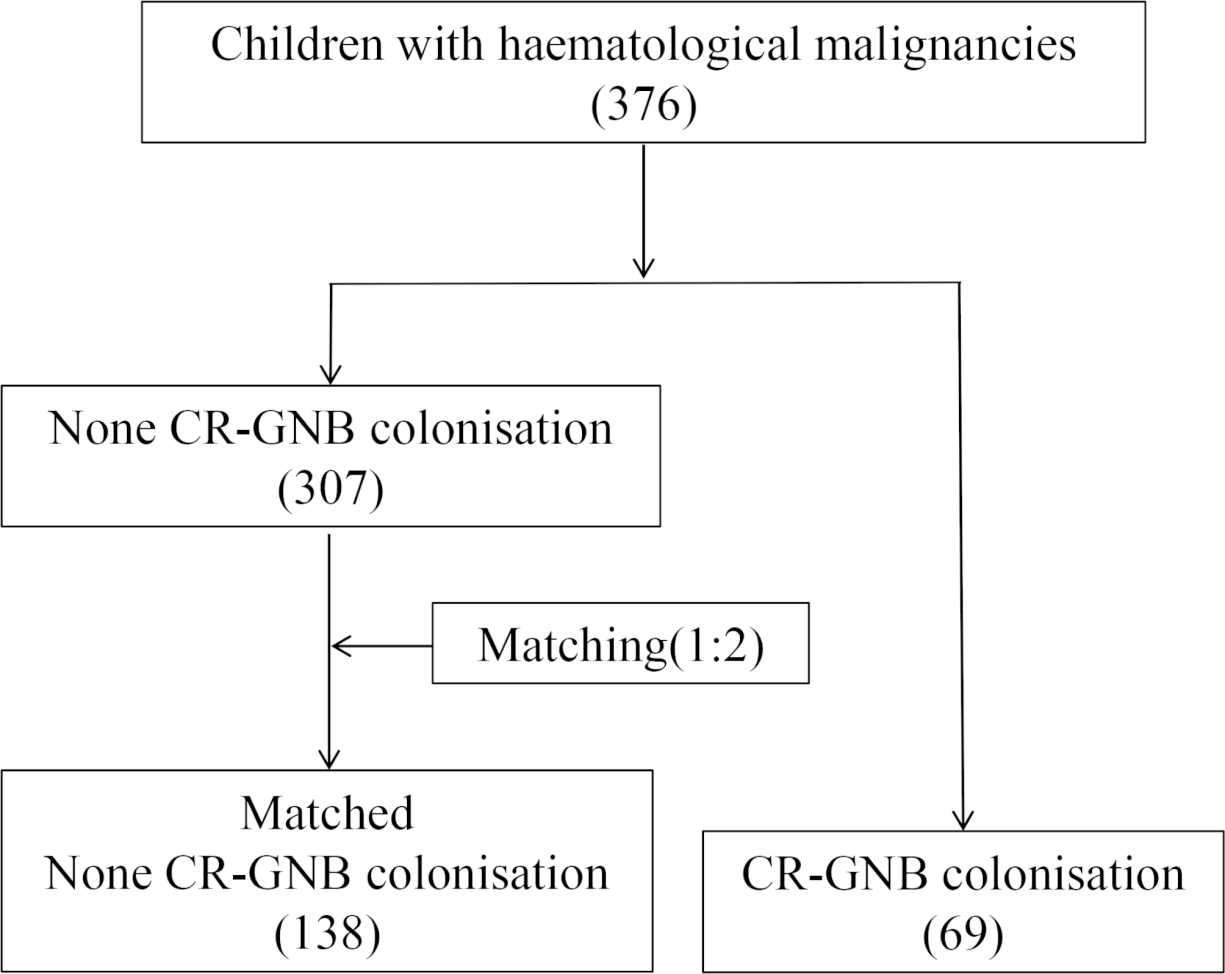
Flow chart of children with haematological malignancies included

The hospitalisation date had the greatest effect on the children’s hospital environment because of the number of admissions, type of patients and season changes over time. Therefore, the different hospitalisation dates will put children in different hospitalisation settings. Thus, the remaining 307 children without CR-GNB colonisation were sorted in chronological order of admission. According to the principle of close hospitalisation date, each child with CR-GNB rectal colonisation was matched with four children without CR-GNB colonisation with the closest hospitalisation date (± 14 days). Two children without CR-GNB colonisation were selected from these four children without CR-GNB colonisation by generating two random numbers (ranging from 1 to 4). Finally, 207 children were included in the study, and this met the requirements for sample size estimation. The flow chart of children with HMs included in the study is illustrated in Fig. 1.***Statistical analysis***.

IBM SPSS Statistics for Windows version 19.0 (IBM Corp, Armonk, NY, USA) was used for data analysis. Continuous variables were expressed as median (interquartile range). The Mann–Whitney U test was used to compare the difference in continuous variables. Differences between categorical variables were analysed using the chi-square test. Conditional logistic regression analysis was used to compute crude odds ratios (cOR) and adjusted odds ratios (aOR) with 95% confidence intervals (CIs). Based on the results of the univariate analysis, variables with a P-value of < 0.1 were included in the conditional logistic regression analysis. The optimal model was determined by establishing multiple models and comparing the Akaike information criterion (AIC) value and likelihood ratio test results (P < 0.05) between each model. The conditional logistic regression analysis was conducted using R version 3.5.3 (R Development Core Team 2019). Statistical significance was defined as P < 0.05, and all tests of significance were two-sided.**Results**.

**Fig. 2:**
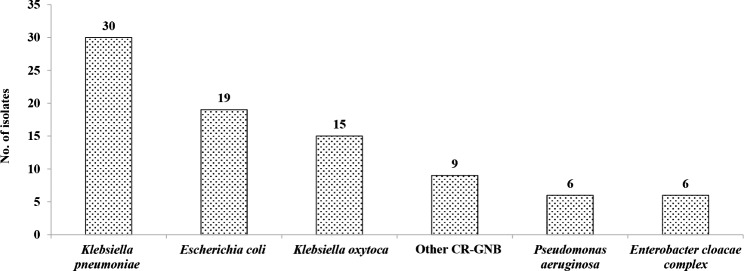
Distribution of carbapenem-resistant Gram-negative bacilli

During the study period, a total of 376 children with HMs underwent rectal CR-GNB tests, and 85 non-duplicated CR-GNB isolates were collected from the rectal swab samples of 69 children with HMs, and the rate of CR-GNB rectal colonisation was 18.4%. More than one CR-GNB was isolated in 13 of 69 children with CR-GNB colonisation, and only one CR-GNB was isolated from the remaining 56 children. From a total of 85 CR-GNB isolates, 30 (35.3%) contained *Klebsiella pneumoniae*; 19 (22.4%), *Escherichia coli*; and 15 (17.6%), *K. oxytoca*. *K. pneumoniae*, *E. coli* and *K. oxytoca* were the leading CR-GNB members. The number of other isolates was less than 10, and the distribution of CR-GNB in this study is shown in Fig. 2.

The median age of children with CR-GNB colonisation was 61.0 (25th–75th percentiles, 38.5–132.0) months, and no significant difference was found when compared with the control group with 72.0 (48.0–132.0) months (P = 0.739). In the CR-GNB colonisation group, 60.9% (42/69) were male, which was not significantly different from that in the control group (58.0%; 80/138, P = 0.689). Moreover, 65 children in the CR-GNB colonisation group (94.2%) had acute myeloid leukaemia (AML) or acute lymphoblastic leukaemia (ALL) as the underlying disease, which was significantly higher than that in the control group (n = 112 [81.2%], P = 0.012). In addition, 66 children in the CR-GNB colonisation group (95.7%) had prior concomitant infections within the last 1 month, which was significantly higher than that in the control group (n = 114 [82.6%], P = 0.009). Fifty children in the CR-GNB colonisation group (72.5%) had prior carbapenem use within the last 1 month, which was significantly higher than that in the control group (n = 68 [49.3%], P = 0.001). This phenomenon was not found in the analyses of other antibiotics. Eleven children (15.9%) in the CR-GNB colonisation group had prior haematopoietic stem-cell transplantation (HSCT), which was significantly higher than that in the control group (n = 4 [2.9%], P = 0.001). In addition, no significant differences were found in other clinical characteristics between the CR-GNB colonisation group and the control group. By comparing the outcomes, eight children in the CR-GNB colonisation group (11.6%) had CR-GNB infections, which was significantly higher than that in the control group (n = 3 [2.2%], P = 0.012). Finally, the 30-day mortality of the CR-GNB colonisation group (n = 4 [5.8%]) was also significantly higher than that of the control group (n = 0 [0.0%], P = 0.020). The demographic characteristics of the children in this study are shown in Table 1.Based on the results of the univariate analysis, AML/ALL, prior concomitant infections within the last 1 month, sepsis, oral mucositis, prior carbapenem use within the last 1 month, prior macrolide use within the last 1 month and prior HSCT were included in the analysis (P < 0.1), and their cOR values are presented in Table 2. By comparing the AIC values and likelihood ratio test results of each model, AML/ALL, prior concomitant infections within the last 1 month, prior carbapenems use within the last 1 month and prior HSCT were included in the final model. Finally, independent risk factors for CR-GNB rectal colonisation in children with HMs included AML/ALL (aOR = 6.84; 95% CI = 1.86–25.20), prior concomitant infections within the last 1 month (aOR = 4.16; 95% CI = 1.17–14.84), prior carbapenem use within the last 1 month (aOR = 2.33; 95% CI = 1.16–4.69) and prior HSCT (aOR = 7.46; 95% CI = 1.81–30.67). The independent risk factors for CR-GNB rectal colonisation in children with HMs are shown in Table 2.**Discussion**.

**Table 1:** Demographic characteristics of the children analysed in this study

Variable	CR-GNB^1^ colonisation group (n = 69)	Control group (n = 138)	χ^2^	*P*-value
Age in months (median, range)	61.0(38.5–132.0)	72.0(48.0-132.0)	/	0.739
Gender (male), n (%)	42(60.9)	80(58.0)	0.160	0.689
AML/ALL^2^, n (%)	65(94.2)	112(81.2)	6.135	**0.012**
Prior concomitant infections within the last 1 month, n (%)	66(95.7)	114(82.6)	6.900	**0.009**
Sepsis	58(84.1)	101(73.2)	3.051	0.081
Respiratory tract infection	34(49.3)	52(37.7)	2.546	0.111
Enteritis	8(11.6)	16(11.6)	0.000	1.000
Oral mucositis	9(13.0)	8(5.8)	3.204	0.073
Prior β-lactam-β-lactamase inhibitor combinations usage within the last 1 month, n (%)	21(30.4)	52(37.7)	1.058	0.304
Prior cephalosporins usage within the last 1 month, n (%)	13(18.8)	31(22.5)	0.361	0.548
Prior 3rd generation cephalosporins usage within the last 1 month, n (%)	13(18.8)	30(21.7)	0.235	0.628
Prior carbapenems usage within the last 1 month, n (%)	50(72.5)	68(49.3)	10.092	**0.001**
Prior macrolides usage within the last 1 month, n (%)	3(4.3)	17(12.3)	3.349	0.067
Prior sulfonamides usage within the last 1 month, n (%)	19(27.5)	34(24.6)	0.203	0.652
Number of days since last chemotherapy(median, range)	15.0(12.0–29.0)	13.5(10.0-23.3)	/	0.116
Prior HSCT^3^, n (%)	11(15.9)	4(2.9)	11.644	**0.001**
WBCs, ×10^9^/L (median, range)	0.55(0.22–2.05)	0.82(0.29–3.12)	/	0.142
Platelets, ×10^9^/L (median, range)	44(17–72)	40(17–127)	/	0.619
CRP, mg/L (median, range)	8.81(0.97–34.98)	5.36(0.50-20.23)	/	0.124
Agranulocytosis	51(73.9)	89(64.5)	1.865	0.172
Outcomes, n (%)				
CR-GNB^3^ infections	8(11.6)	3(2.2)	6.349	**0.012**
Thirty-day mortality	4(5.8)	0(0.0)	5.385	**0.020**

^1^ CR-GNB: Carbapenem-resistant Gram-negative bacillus; ^2^ AML: Acute myeloid leukemia/ALL: Acute lymphoblastic leukemia; ^3^ HSCT: Haematopoietic stem-cell transplantation. Statistically positive significant results at the 5% level (*P* < 0.05) are indicated in bold

**Table 2:** Independent risk factors for rectal colonisation with carbapenem-resistant Gram-negative bacillus in children with haematological malignancies – conditional logistic regression

Variables	cOR^3^ (95% CIs^4^)	aOR^5^ (95% CIs)	*P*-value
AML/ALL^1^	4.15(1.36–12.68)	6.84(1.86–25.21)	**0.004**
Prior concomitant infections	4.95(1.41–17.34)	4.16(1.17–14.84)	**0.028**
Sepsis	2.03(0.94–4.41)	/	/
Oral mucositis	2.39(0.88–6.49)	/	/
Prior carbapenems usage within the last 1 month	2.54(1.37–4.73)	2.33(1.16–4.69)	**0.018**
Prior macrolides usage within the last 1 month	0.31(0.08–1.11)	/	/
Prior HSCT^2^	6.87(1.90-24.77)	7.46(1.81–30.67)	**0.005**

^1^ AML: Acute myeloid leukemia/ALL:Acute lymphoblastic leukemia. ^2^ HSCT: Haematopoietic stem-cell transplantation. ^3^ cOR: Crude odds ratios. ^4^ CIs: Confidence interval. ^5^ aOR: Adjusted odds ratios. Statistically positive significant results at the 5% level (*P* < 0.05) are indicated in bold

CR-GNB infections seriously threaten the survival of patients, especially those with chronic granulocytopenia after chemotherapy, in HM treatment units [16–19]. CR-GNB intestinal colonisation is an important factor in CR-GNB infection and related deaths because the intestinal microbiota provides a perfect environment for interspecies drug resistance gene transfer [20].

Therefore, CR-GNB intestinal colonisation in patients with HMs, especially in children, must be studied to prevent CR-GNB infections. This case–control study was conducted to identify the risk factors for CR-GNB rectal colonisation in children with HMs. The rate of CR-GNB rectal colonisation reached 18.4% during the study period, an alarming finding. The top three bacteria were all Enterobacteriaceae (K. pneumoniae, E. coli and K. oxytoca) and accounted for three-quarters of CR-GNB isolates. This suggests that Enterobacteriaceae is the main force of CR-GNB colonisation in children with HMs, which may be because Enterobacteriaceae is the main component of gut microbiota. From the demographic characteristics, no significant difference in age and sex was found between the CR-GNB colonisation group and the control group, which indicated that the two groups were relatively matched in terms of grouping. CR-GNB rectal colonisation significantly affected the outcomes of children with HMs. Significant differences were found in both CR-GNB infections and 30-day mortality between the two groups of children with HMs. In other words, the incidence of CR-GNB infections and 30-day mortality were significantly higher in the CR-GNB colonisation group than in the control group. However, this significant difference may be underestimated because clinicians may have adopted more aggressive management measures to prevent CR-GNB infections after receiving news of CR-GNB rectal colonisation, which may have prevented some cases from progressing to CR-GNB infections or even death. The incidence of CR-GNB infections and the 30-day mortality in the CR-GNB colonisation group were higher than those calculated in this study if clinicians did not adopt more aggressive management measures.

The logistic regression analysis showed that AML/ALL, prior concomitant infections within the last 1 month, prior carbapenem use within the last 1 month and prior HSCT are associated with an increased risk of rectal colonisation with CR-GNB in children with HMs. In children with AML or ALL, the immune balance is often disrupted, and this disruption is exacerbated by agranulocytosis caused by chemotherapy. The incidence of infectious diseases in children with AML or ALL will be far greater than that in other populations, and the use of antibiotics will be unavoidable. Frequent, heavy and persistent use of antibiotics, especially carbapenems, leads to further screening for CR-GNB. Currently, HSCT is a curative option for many HMs in children; however, the probability of infection will be greatly increased during immunosuppression and even lead to death [21, 22]. The increased occurrence of infectious diseases necessarily leads to the increased use of antibiotics. This may lead to a further selection of resistant bacteria, thus allowing the emergence of resistant bacteria as dominant bacteria. In addition, the use of prophylactic antibiotics during immunosuppression to prevent infectious diseases may lead to similar results. In summary, these risk factors are basically related to the prevention or treatment of infectious diseases. This has led to an increase in the use of antibiotics, especially carbapenems, which help in screening for resistant bacteria and ultimately lead to CR-GNB colonisation. Therefore, we believe that the use of antibiotics, especially carbapenems, is essential for the treatment of CR-CNB colonisation in children with HMs. This conclusion is consistent with the results of Chiotos et al. [14] Although they did not limit their study population to children with MHs and did not distinguish colonisation from infection, their conclusions similarly highlighted antibiotic use as an essential risk factor. Given that antibiotic use is firmly associated with CR-GNB colonisation, screening for CR-GNB colonisation should not be limited to children with MHs. Screening for CR-GNB colonisation is necessary for children with frequent or high-dose antibiotic use, especially those from intensive care units or with immunodeficiency caused by other factors.

The limitations of this study should be considered. First, As this was a retrospective case-control study, resistance genes were not analyzed. Second, As a single-center study, the conclusion might not apply to other hospitals because the cases included were all solely from the same hospital Third, the sample size was limited because the patients were from the same ward of the same hospital, and the study period was limited. Although the requirements for sample size estimation were met, the representativeness of the study results may be limited. Additional study with expanded scope and large sample sizes is needed. Finally, further subgroup analysis is needed because of differences in the mechanisms by which Enterobacteriaceae and Pseudomonas develop resistance. However, given the limited sample size of other GNBs, further subgroup analyses were not included in this study.

## Conclusions

The risk factors for CR-GNB rectal colonisation in children with HMs include AML/ALL, prior concomitant infections within the last 1 month, prior carbapenems usage within the last 1 monthand prior HSCT. Therefore, clinicians should strengthen the detection of CR-GNB rectal colonisation in children with HMs and adjust the management measures to prevent CR-GNB infections promptly in the presence of the four risk factors, such as strengthening the monitoring of infectious indicators and adjusting the use of prophylactic or therapeutic antibiotics.

## Data Availability

The datasets used and/or analysed during the current study are available from the corresponding author on reasonable request.
